# Assessment of arterial stiffness, oxidative stress and inflammation in acute kidney injury

**DOI:** 10.1186/1471-2369-10-15

**Published:** 2009-06-18

**Authors:** Robert G Fassett, Vincent D'Intini, Helen Healy, John Gowardman, Jay-Sen Gan, James E Sharman, Jeff S Coombes

**Affiliations:** 1 Renal Medicine, Royal Brisbane and Women's Hospital, Brisbane, Queensland, Australia; 2 Intensive Care Unit, Royal Brisbane and Women's Hospital, Brisbane, Queensland, Australia; 3 Department of Nephrology, Royal Hobart Hospital, Hobart, Tasmania, Australia; 4 Menzies Research Institute, University of Tasmania, Hobart, Tasmania, Australia; 5 School of Human Movement Studies, The University of Queensland, Brisbane, Queensland, Australia; 6 School of Medicine, The University of Queensland, Brisbane, Queensland, Australia

## Abstract

**Background:**

It is well know that arterial stiffness, oxidative stress and inflammation are features of chronic kidney disease. The arterial changes have a multitude of potential interconnected causes including endothelial dysfunction, oxidative stress, inflammation, atherosclerosis and vascular calcification. There is evidence that arterial stiffness becomes progressively worse as CKD progresses. The contribution of the biochemical changes of uremic toxicity to arterial stiffness is less clear. The aim of this study is to elucidate the vascular changes in acute kidney injury. We hypothesise that arterial stiffness will be increased during acute kidney injury and this will return to normal after kidney function recovers.

**Methods/Design:**

One hundred and forty four patients with acute kidney injury defined as an acute increase in serum creatinine to > 133 μmol/l or urea > 14.3 mmol/l or urine output < 410 ml/day will be recruited. Baseline measures of aortic pulse wave velocity, augmentation index, and brachial and central blood pressure will be recorded along with blood measures for oxidative stress and inflammation. Repeat measures will be taken at six and 12 months after the onset of the acute kidney injury.

**Discussion:**

The role and contribution of the biochemical changes to arterial stiffness in the acute phase of kidney disease is not known. This study will primarily assess the time course changes in pulse wave velocity from the onset of acute kidney injury and after recovery. In addition it will assess augmentation index, central blood pressure and oxidative stress and inflammation. This may shed light on the contribution of biochemical kidney toxins on arterial stiffness in both acute kidney injury and chronic kidney disease.

**Trial Registration:**

**ACTRN **12609000285257

## Background

Vascular disease, characterised by premature atherosclerosis and large artery stiffness, is a feature of advanced chronic kidney disease (CKD) [1,2]. Central pulse pressure, augmentation index (AIx) and aortic pulse wave velocity (PWV) are all predictors of morbidity and mortality in patients with CKD [1,3,4]. Large artery stiffness may also be related to the progression of CKD. The mechanisms and time frames for the development of arterial stiffness are unclear but may include traditional cardiovascular risk factors such as dyslipidemia and hypertension as well as non-traditional risk factors such as oxidative stress and inflammation. Moreover, to our knowledge no studies have assessed the relationship between arterial stiffness in the uraemic environment of acute kidney injury. Measuring arterial stiffness in this setting would enable us to determine whether changes in arterial stiffness occur due to the biochemical changes associated with acute kidney injury without the interference of factors such as chronic vascular calcification that exist in chronic kidney disease. This may in turn help to understand the potential for reversibility of uremic biochemical effects on arterial stiffness.

## Methods/Design

### Study Design and Setting

This is a longitudinal observational clinical study. Patients from the Royal Brisbane and Women's Hospital and the Launceston General Hospital will be recruited into this study. These hospitals service populations of approximately 1.2 million and 250,000 respectively.

### Ethical Considerations

The Tasmanian Statewide Scientific and Ethics Committees and the Human Research and Ethics Committee of the Royal Brisbane and Women's Hospital have approved this study.

### Identification of Eligible Patients

#### Study population

One hundred and forty four patients with acute kidney injury and 45 matched controls.

Acute kidney injury participants will be recruited from two settings at each hospital.

1. The Intensive care unit: The distinguishing features of intensive care research are the requirements to recruit and obtain informed consent rapidly when it may be difficult to communicate with the patient. As the patient requirements for this study are minimal and non-invasive it is not expected that these requirements will be problematic. All attempts will be made to gain informed written consent directly from the patient. The patients will be approached by one of the investigators who will explain the study, provide them with the information sheet and request they consider entering the study and therefore sign the consent form. If patients are unable to provide written informed consent themselves (e.g. they are unconscious or too unwell) the next of kin will be approached by one of the investigators who will explain the study, provide them with the information sheet and request that they consider the patient enters the study and sign the consent form on their behalf.

2. The Renal medicine service: This will include the renal inpatients and referrals from General Internal Medicine, Surgery and other departments in the Royal Brisbane and Women's Hospital. For these patients the recruitment will be based on the standard informed consent process as described above for the conscious intensive care unit patient.

#### Accrual time

Two years

### Eligibility

**Figure 1:**
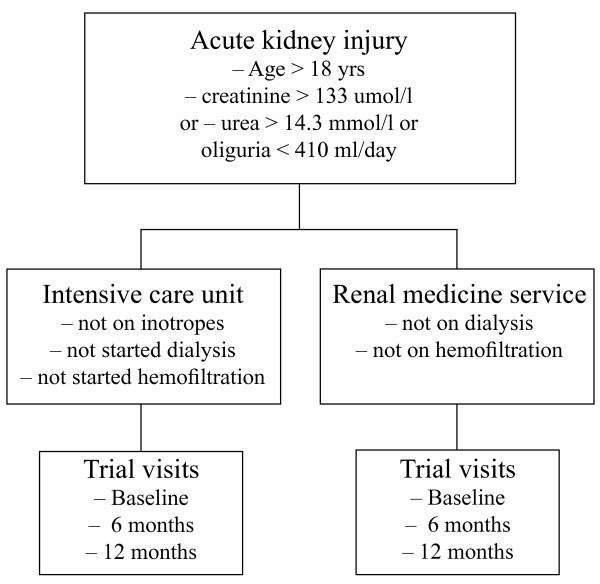
**Study patient flow**.

Inclusion criteria: Male and female patients > 18 years of age presenting to the Royal Brisbane and Women's Hospital and the Launceston General Hospital with acute kidney injury but not having started dialysis or continuous venovenous hemofiltration will be eligible for recruitment (Figure 1). Acute kidney injury will be defined as the presence of a serum creatinine > 133 μmol/l or urea > 14.3 mmol/l or urine output < 410 ml/day [5,6].

Exclusion criteria: Patients receiving vasoactive drugs such as inotropic agents or on dialysis or continuous venovenous hemofiltration will be excluded. In addition, any patients with a condition the treating doctor deems to preclude the patient participating based on safety concerns will be excluded. Patients planned for palliative care, unwilling to comply with trial requirements, or whose life expectancy is severely limited (< six months) due to pre-existing malignancy or other disease will be excluded. Finally, patients that are pregnant or have a planned pregnancy will also be excluded.

### Primary Objectives and Primary Outcome Measures

#### Aims and hypotheses

##### Primary aim

To determine the effects of acute kidney injury on arterial stiffness

##### Hypothesis 1

Patients with acute kidney injury will have increased arterial stiffness compared to matched controls.

##### Hypothesis 2

Patients who recover kidney function following acute kidney injury will have a concomitant reduction in arterial stiffness.

#### Primary outcome measure

##### Arterial stiffness

Carotid to femoral PWV will be derived by electrocardiography-gated sequential applanation tonometry (SPT-301 Mikro-Tip, Millar Instruments, Houston, Texas) using the foot-to-foot method (SphymoCor™ 7.01 AtCor, Sydney, Australia) [7]. Testing will be performed in a temperature-controlled room and after 5–10 minutes rest with the subject in the supine position prior to the first measure. Two measures will be averaged for the estimation of aortic PWV. Aortic stiffness (carotid to femoral PWV) will be derived by electrocardiography-gated sequential applanation tonometry using the foot-to-foot method (SphygmoCor 7.01 AtCor, Sydney, Australia) as previously described [7]. Briefly, three ECG electrodes are placed on the patient's chest and they are required to lie quietly for 15 minutes. Brachial blood pressure is measured and then the tonometer is applied sequentially over the radial, carotid and femoral arteries. The procedure takes around 15 minutes, but this may vary depending on patient morphology.

### Secondary Aim and Secondary Outcome Measures

#### Secondary Aim

To determine the associations between oxidative stress and inflammation with changes in arterial stiffness and central blood pressure in patients with acute kidney injury.

#### Hypothesis

There will be significant relationships between oxidative stress and inflammation and changes in arterial stiffness and central blood pressure in patients with acute kidney injury.

### Secondary outcome measures

#### Oxidative Stress

The outcome measure for oxidative stress will be plasma isoprostanes measured using gas chromatography mass spectrometry [8]. In addition, plasma protein carbonyls, total antioxidant capacity and antioxidant enzyme activities will also be measured to further assess oxidative stress and antioxidant status.

#### Inflammation

The outcome measure for inflammation will be pentraxin 3 measured using an ELISA assay [9]. In addition, interleukin (IL)-6, IL-8, IL-10, high sensitivity C reactive protein and tumour necrosis factor-alpha will be measured as further markers of inflammation.

#### Central blood pressure and augmentation index (AIx)

The SphygmoCor device will be used to determine central blood pressure and AIx as additional markers of arterial stiffness. A central (ascending aortic) pressure waveform will be derived by radial tonometry as previously described [7]. This method involves placing a tonometer over the radial artery and using a generalised transfer function to transform the radial pressure waveform. Central pulse pressure will be recorded as an estimate of afterload and AIx as a composite measure of systemic arterial stiffness and arterial wave reflection. The central waveform will be calibrated by the average of two measures of brachial blood pressure using a semi-automated device (UA-767, A&D, Saitama, Japan) after five minutes of supine rest.

#### Additional data and routine measures

Routine measures including full blood count, electrolytes, coagulation profile, liver function test and lipid profile will be carried out at the Hospitals' Pathology Laboratories. Other data collected at the time will include demographic details, medical history and medication profile.

#### Sample handling and storage

Upon collection, blood samples will be placed on ice and then centrifuged (500 g) at 4°C for five minutes. One ml aliquots of plasma will be stored in eppendorf tubes immediately at -80°C for future biochemical analyses of oxidative stress and inflammation.

### Baseline testing

After patients provide informed consent they will undergo baseline testing to measure resting peripheral and central blood pressure, arterial stiffness and have blood taken for standard biochemistry and measures of oxidative stress and inflammation. Control data will be obtained from a database of one of the authors that contains arterial stiffness measures on healthy individuals with normal kidney function. Subjects from this database will be selected to match with patients included in the analyses.

### Visits two and three

All measures taken at baseline will be repeated at six and twelve months after the onset of the acute kidney injury.

### Recovery of kidney function

This will be defined as return of kidney function to normal (eGFR > 60 ml/min/1.73 m^2^) or, where data is available, a return to the level of kidney function within 5% assessed by eGFR present within three months prior to the acute kidney injury.

### Withdrawal from Study

If requested, subjects will be withdrawn from the study without prejudice, as documented and explained at the time of consenting.

### Statistical considerations

#### Sample size calculation

Sample sizes of 144 acute kidney injury patients and 45 matched controls have been determined as follows. The primary outcome measure is aortic PWV. To our knowledge, there is no published aortic PWV data available for acute kidney injury patients. We therefore used haemodialysis patient aortic PWV data from one of our other investigations, the PEDAL study[10]. We found that the aortic PWV of dialysis patients was 9.4 +/- 1.9 m/sec. Previous work from Blacher et al reported that for each 1 m/sec increase in aortic PWV there is a 34% increase in both cardiovascular and total mortality [11]. We assume that this increase is clinically relevant. Therefore in order to detect a 1 m/sec difference in PWV compared to controls (hypothesis 1) or 1 m/sec change in aortic PWV in the patients (hypothesis 2) 45 patients will be required (beta 1-0.8 = 0.20, alpha = 0.05 using a two tailed paired t test). This assumes the initial aortic PWV will be 9.4 +/- 1.9 m/sec and if the hypotheses are supported the aortic PWV in controls and on restoration of kidney function will be 8.4 +/- 1.9 m/sec. However allowing for 1) a high loss to follow-up rate (50%) because of the mortality associated with acute kidney injury, particularly in ICU, and 2) the expected number of patients who will recover kidney function after 12 months (60%) then at least 144 patients will need to be recruited to achieve the 45 patients required.

### Statistical analyses

General linear modelling will be used to test the hypotheses. Bi-variate and multivariate techniques will be used to assess the secondary aims of the relative independent effects of variables on the outcomes measures. Significance will be assumed if P < 0.05.

## Discussion

The objective of this study is to assess whether the biochemical changes seen in acute kidney injury can affect arterial stiffness. Although arterial stiffness is a feature of vascular dysfunction in chronic kidney disease it is not clear how much, if at all the biochemical abnormalities seen in the circulation account for such changes. In addition, the study will assess augmentation index, central blood pressure and oxidative stress and inflammation. Data from this study may lead to a better understanding of the vascular dysfunction seen in kidney disease.

## Competing interests

The authors declare that they have no competing interests.

## Authors' contributions

RGF and JSC designed the study and wrote the protocol. JG provided advice and input and will be involved with the intensive care component of the study. VD and HH provided advice and input and will be involved with the renal medicine department component of the study. J-S G will perform arterial stiffness measures and contributed to manuscript review. JS provide advice and input into the arterial stiffness measures and reviewed the manuscript. All authors read and approved the final manuscript.

## Pre-publication history

The pre-publication history for this paper can be accessed here:

http://www.biomedcentral.com/1471-2369/10/15/prepub
